# Nurturing Movement: Longitudinal Associations Between Caregiver Type, Adolescent Diet, and Young Adult Physical Activity in a National Cohort

**DOI:** 10.3390/nu17111874

**Published:** 2025-05-30

**Authors:** Rahel Mathews, Danielle K. Nadorff, Riley Cowart

**Affiliations:** 1 Department of Food Science, Nutrition and Health Promotion, Mississippi State University, Starkville, MS 39762, USA; rm933@msstate.edu; 2 Department of Psychology, Mississippi State University, Starkville, MS 39762, USA; 3 Mississippi State University, Starkville, MS 39762, USA; rec442@msstate.edu

**Keywords:** grandparents, nutrition, physical activity, longitudinal studies, adolescent health

## Abstract

Background: Childhood obesity rates remain high in the United States, with long-term implications for physical and mental health. Emerging research suggests that caregiving arrangements, particularly those involving non-parental caregivers such as grandparents, may influence adolescent health behaviors, including diet and physical activity. This study examined whether caregiver type (parents-only, grandparents-only, or multi-generational households) during adolescence predicted dietary patterns and physical activity trajectories into young adulthood. Methods: Data were drawn from 6260 participants in the National Longitudinal Study of Adolescent to Adult Health (Add Health). Caregiver type was identified at baseline (Wave 1), and dietary intake and physical activity were assessed across four waves from adolescence (~age 15) to early adulthood (~age 29). We employed generalized linear models and linear mixed-effects models with multiply imputed data to examine changes in sedentary behavior, individual exercise, and team sport participation over time, controlling for age, sex, and race. Results: Overall dietary intake at baseline did not significantly differ by caregiver type (Wilks’ Λ = 0.998, *p* = 0.389); however, an exploratory comparison indicated lower dairy consumption in homes with a grandparent present (*t*(6258) = 1.995, *p* = 0.046). Trajectories of individual exercise differed significantly by caregiver type over time (Time × Caregiver interaction: *F*(6, 10,395.601) = 2.795, *p* = 0.010), with adolescents in grandparent-only households reporting higher individual exercise at Wave 1. Sedentary behavior trajectories also significantly differed by caregiver group over time (Wave × Caregiver interaction: *F*(6, 18,951.310) = 23.026, *p* < 0.001). Baseline nutrition was positively associated with individual exercise trajectories into young adulthood (Time × Nutrition interaction: *F*(2.961, 13,096.103) = 3.974, *p* = 0.012). Conclusions: Caregiver structure during adolescence appears to shape long-term physical activity patterns, albeit modestly. These findings highlight the need to consider diverse family configurations—particularly grandparent-led households—when designing public health interventions to promote adolescent nutrition and physical activity.

## 1. Introduction

Regular physical activity and adequate nutrition are fundamental to children’s holistic growth and enduring well-being. Early childhood physical activity is consistently linked to a spectrum of benefits, including enhanced motor skills, cognitive development, bone health, and favorable cardiometabolic profiles [1]. At the same time, sufficient macro- and micronutrient intake is crucial not only for neurological development, but also for numerous physiological processes essential for overall systemic health, including growth, immune function, and energy production [2]. Furthermore, as dietary habits established early often persist into adulthood [3], fostering healthy eating and activity behaviors from a young age is critically important.

The persistence of unhealthy dietary and activity patterns from childhood into adulthood carries significant public health implications, particularly given current global health trends. In 2022, an estimated 160 million children and adolescents (aged 5–19 years) worldwide were living with obesity, and 390 million were overweight—a near quadrupling in obesity prevalence for this age group since 1990 [4]. This is particularly concerning as childhood obesity strongly predicts adult obesity and related chronic diseases. Furthermore, in the United States (US), physical activity levels among youth remain low; 2022–2023 data show only about 19.5% of children aged 6–17 meet daily recommendations [5]. This lack of activity is frequently accompanied by high levels of sedentary screen time. For instance, data from 2021–2023 show that approximately half (50.4%) of US teenagers (aged 12–17) engaged in four or more hours of daily screen time [6], while estimates for children aged 8–12 suggest average screen use of 4–6 h daily [7]. Addressing these interconnected behaviors requires a deeper understanding of their developmental roots.

Bronfenbrenner’s socioecological model [8] offers a valuable framework for understanding how health behaviors are shaped by multiple levels of influence, with the microsystem—the child’s immediate environment—being particularly formative. More recent applications of this model continue to underscore its relevance in examining how family and caregiving contexts affect child development and health outcomes [9]. Within this microsystem, caregivers are paramount, acting as “nutritional gatekeepers” who significantly influence children’s dietary intake by controlling the food purchased, prepared, and served [10]. They also model crucial eating behaviors, such as portion control, food preferences, and responses to hunger and satiety cues [3]. For instance, the establishment of family mealtime routines, a key microsystem interaction, is consistently linked with healthier dietary patterns, and shared meals in childhood and adolescence often predict better dietary quality, including higher fruit and vegetable consumption, in later years [11]. Similarly, caregivers profoundly shape children’s physical activity through multiple pathways, including providing direct support, modeling active lifestyles themselves, and expressing enjoyment in physical pursuits [12]. Consequently, variations in caregiver arrangements—such as those involving parents, grandparents, or multi-generational configurations—create distinct microsystem dynamics that can differentially impact children’s health trajectories.

Family structures in the US are diverse and evolving. Notably, grandparents are increasingly involved in raising grandchildren, either as primary caregivers or in multi-generational households [13]. This demographic shift necessitates a closer look at how these specific caregiver configurations influence child health. While grandparents offer vital support, research yields mixed results regarding their impact on child weight, diet, and activity, potentially due to differing socioeconomic factors, health knowledge, or generational norms.

Much existing research is cross-sectional, limiting understanding of long-term impacts. Longitudinal studies are needed to examine how different family microsystems during adolescence influence both nutrition and physical activity development across the transition to adulthood. This study utilizes the National Longitudinal Study of Adolescent to Adult Health (Add Health) to address this gap. Guided by the socioecological model [8,9], we investigate how adolescents’ caregiver type (parents-only, grandparents-only, and multi-generational) relates to baseline nutritional intake (Wave 1) and trajectories of physical activity (sedentary activities, individual exercise rates, and team sports participation levels) from adolescence through young adulthood (Waves 1–4), considering the chronosystem aspect of development.

### 1.1. Influence of Caregiver Type on Nutrition

The influence of differing caregiver configurations, particularly those involving grandparents, on children’s nutrition is complex and multifaceted. While some research links grandparent involvement with potentially less favorable dietary outcomes, such as indulgent feeding, increased risk of overweight/obesity, or higher BMI z-scores [14,15,16,17], other studies indicate that grandparents can also promote healthy eating behaviors [16]. This variability suggests that the nutritional impact within grandparent-involved microsystems likely differs based on individual caregiver beliefs, family dynamics, and socioeconomic factors, highlighting the need for further in-depth investigation.

### 1.2. Influence of Caregiver Type on Physical Activity and Sedentary Behavior

Similarly, the role of caregiver type in shaping children’s physical activity and sedentary behaviors presents a mixed picture. Some studies associate grandparent involvement with less favorable patterns, such as increased screen time [7,18,19,20,21], while other research indicates that children in grandparent-led or multi-generational homes may achieve higher rates of daily physical activity [18]. The nature and intensity of activity engagement can also depend on factors like the caregiver’s own mobility and access to resources [19], again pointing to the complexity of these influences.

### 1.3. Socioeconomic Context and Longitudinal Health Impacts

It is also important to consider the broader socioeconomic context (exosystem) influencing these family microsystems. Grandparent-headed households, for example, are often reported to face greater socioeconomic disadvantages, which can constrain access to resources pivotal for healthy lifestyles, such as participation in organized sports [20,22,23,24].

Ultimately, the health behaviors established during adolescence within these varied family environments have profound and lasting implications (chronosystem). For instance, physical activity levels in youth are linked to health-related quality of life [25], and childhood weight status is a strong predictor of adult obesity and related health risks [26,27].

### 1.4. Current Study

The preceding review highlights the critical role of childhood nutrition and physical activity on long-term health and development, alongside persistent concerns regarding obesity and sedentary trends [28,29]. While caregivers are established influencers of these behaviors [30,31,32,33], a nuanced understanding of the long-term impact of different family caregiving structures—particularly those involving grandparents—requires further longitudinal study.

Therefore, the present investigation, guided by Bronfenbrenner’s socioecological model [8], addresses this gap. Utilizing data from the National Longitudinal Study of Adolescent to Adult Health (Add Health), we examine how variations in the adolescent family microsystem, specifically caregiver type (parents-only, grandparents-only, or multi-generational households), relate to (1) adolescent nutritional intake at baseline (Wave 1), and (2) the trajectories of sedentary behavior, individual exercise, and team sports participation as these adolescents transition into young adulthood (Waves 1–4). This longitudinal approach allows for an examination of these influences via the chronosystem over an approximately 14-year period.

This research seeks to make several contributions: (1) extending beyond traditional parental influence to explicitly include grandparent-only and multi-generational contexts; (2) leveraging a national longitudinal dataset (Add Health) to assess how caregiver influence on activity patterns persists into young adulthood; and (3) providing a nuanced understanding by assessing multiple dimensions of physical activity (sedentary activities, individual exercise rates, and team sports participation levels). Ultimately, by exploring the interplay between family structure, adolescent nutrition, and long-term activity, this study aims to inform targeted interventions and policies promoting healthy lifestyles across diverse family contexts.

#### Hypotheses

**Hypothesis** **1**: *There will be significant group differences by caregiver type on childhood nutrition levels at Time 1. Specifically, it is hypothesized that those raised by parents will report higher levels of nutrition than those raised by grandparents or in multi-generational homes.*

**Hypothesis** **2A**: *Caregiver type (parents-only, grandparents-only, or multi-generational homes) will significantly influence adolescents’ time spent in sedentary activities across the four time points. Specifically, it is hypothesized that children from parents-only homes will be less sedentary than those from homes led by a grandparent or multi-generational homes.*

**Hypothesis** **2B**: *Caregiver type (parents-only, grandparents-only, or multi-generational homes) will significantly influence adolescents’ time spent in individual exercise activities across the four time points. Specifically, it is hypothesized that children from parents-only homes will engage in more individual exercise than those from homes led by a grandparent or multi-generational homes.*

**Hypothesis** **2C**: *Caregiver type (parents-only, grandparents-only, or multi-generational homes) will significantly influence adolescents’ time spent in team exercise activities across the four time points. Specifically, it is hypothesized that children from parents-only homes will engage in more team exercise than those from homes led by a grandparent or multi-generational homes.*

**Hypothesis** **3A**: *Childhood nutrition levels at Time 1 will impact sedentary activity levels over time, such that low levels of nutrition will be associated with higher levels of sedentary activities across time.*

**Hypothesis** **3B**: *Childhood nutrition levels at Time 1 will impact individual exercise levels over time, such that low levels of nutrition will be associated with lower levels of individual exercise across time.*

**Hypothesis** **3C**: *Childhood nutrition levels at Time 1 will impact team sport exercise levels over time, such that low levels of nutrition will be associated with lower levels of team sports participation across time.*

## 2. Materials and Methods

### 2.1. Participants and Procedure

Data were taken from the National Longitudinal Study of Adolescent to Adult Health (Add Health [34]). Add Health is a longitudinal study that began in 1994 with a nationally representative sample of adolescents in grades 7–12 in the United States. The study has followed these individuals over five waves of data collection, though the present study utilizes data from the first four waves, spanning 1994–1995 (Wave 1, adolescents aged approximately 15 years) to 2008 (Wave 4, young adults aged approximately 29 years). All survey items used in this analysis were developed and administered by the Add Health research team. The full set of questionnaire instruments and item wordings can be accessed through the Add Health Documentation Navigator at https://addhealth-navigator.cpc.unc.edu, accessed on 27 May 2025.

Add Health employed a multi-stage stratified sampling design to ensure a representative sample of adolescents across the United States [34]. Data collection involved in-school questionnaires (administered to students in grades 7–12 during the 1994–1995 school year) and in-home interviews. Trained field interviewers from the National Opinion Research Center (NORC) at the University of Chicago conducted the Wave 1 and 2 in-home interviews, while RTI International conducted the Wave 3 and 4 in-home interviews [35]. These instruments gathered extensive data on demographics, social and academic experiences, health behaviors, and family life.

This study utilized de-identified, publicly available data from Add Health. Consistent with guidance from the U.S. Department of Health and Human Services Office for Human Research Protections (OHRP), research using such data does not constitute human subjects research requiring Institutional Review Board approval [36].

The initial sample available for this study from the Add Health dataset comprised N = 6504 participants. There were 5800 households headed by parents with no grandparents present, 135 households headed by grandparents with no parents present, and 325 households with both generations present. Due to missing data primarily on the longitudinal physical activity measures (Waves 2–4 for sedentary, individual, and team sports) and for some cases of the Wave 1 caregiver type (*n* = 244 missing original caregiver type), multiple imputation (MI) was performed. Using SPSS Statistics Version 29.0.2.0, twenty imputed datasets (M = 20) were generated via the fully conditional specification (FCS) method (chained equations). The imputation model included all variables used in subsequent analytical models: all sedentary activity (H1DA3, H2DA3, H3DA4, H4DA1), individual exercise (H1DA4, H2DA4, H3DA9, H4DA3), and team sports (H1DA5, H2DA5, H3DA10, H4DA4) measures across Waves 1–4; caregiver group, dichotomized baseline nutrition; the five raw Wave 1 dietary items (H1GH32—H1GH36); and covariates (age at Wave 1 (H1GI1Y), sex (BIO_SEX), and race/ethnicity (H1GI8)). All reported analyses were conducted on the 20 imputed datasets, with results pooled according to Rubin’s rules. The effective sample size for pooled analyses was approximately N = 6496 for Hypothesis 1 and ranged from N ≈ 4703 to N ≈ 4727 for the repeated measures analyses (Hypotheses 2 and 3), reflecting the cases with sufficient data for each specific model after imputation.

The demographic characteristics of the sample included in the imputation (N = 6504) were as follows: 51.6% female; the average birth year was 1980 (range 1974–1983), corresponding to an average age of approximately 15.2 years at Wave 1 (1994–1995) and 28.2 years at Wave 4 (2008); the racial/ethnic composition was 40.1% White, 28.4% Black/African American, 12.9% American Indian/Native American, 8.9% Asian/Pacific Islander, and 9.7% Other/Multiracial.

### 2.2. Measures

Sedentary Activities: Time spent in sedentary activities was assessed across the first four waves of the ADD Health study (Wave 1: H1DA3; Wave 2: H2DA3; Wave 3: H3DA4; Wave 4: H4DA1). Participants were asked to report the number of hours they spent watching television on an average school day and an average weekend day. Responses were collected on a 6-point Likert scale ranging from “None” to “5 or more hours.”

Individual and Team Exercise: Levels of individual and team exercise were assessed across the four waves: individual exercise (Wave 1: H1DA4; Wave 2: H2DA4; Wave 3: H3DA9; Wave 4: H4DA3), and team sports (Wave 1: H1DA5; Wave 2: H2DA5; Wave 3: H3DA10; Wave 4: H4DA4). Similarly to sedentary activities, participants were asked to report the number of hours they spent on each activity on an average school day and an average weekend day. Responses were collected on a 6-point Likert scale ranging from “None” to “5 or more hours.”

Dietary Habits: Several dietary habits were assessed at Wave 1 of the study. Participants were asked if they ate food from the following categories yesterday:

- Dairy products (H1GH32): Milk, cheese, yogurt, etc.
- Fruits and juice (H1GH33): Oranges, apples, bananas, fruit juice, etc.
- Vegetables (H1GH34): Green salad, carrots, potatoes, etc.
- Bread (H1GH35): White bread, rolls, tortillas, etc.
- Desserts (H1GH36): Cake, cookies, ice cream, etc.

Responses were collected on a 3-point Likert scale ranging from “Didn’t eat” to “Ate twice or more.” From these items, a Healthy Diet Score variable was created by summing the responses for dairy products (H1GH32), fruits and juice (H1GH33), and vegetables (H1GH34). This sum score could range from 0 to 6. This score was then dichotomized. Based on the distribution of the Healthy Diet Score, a cut-point of ≤3 was used to classify participants into a “Low Nutrition” group (coded 0), and a score of >3 was used to classify participants into a “High Nutrition” group (coded 1). This categorization resulted in approximately 47.4% of the sample in the “Low Nutrition” group and 52.6% in the “High Nutrition” group.

Control Variables: Several control variables were included in the study:

- Age: Age at Wave 1 was measured by birth year (H1GI1Y).
- Sex: Sex was coded as male or female (BIO_SEX).
- Race: Race was assessed using a multi-category variable (H1GI8). Categories included White, Black or African American, American Indian or Native American, Asian or Pacific Islander, and Other.

Family Structure: Participants were classified into three groups based on their living arrangements at Wave 1:

- Parent-headed households (coded 1): Participants living with at least one biological or adoptive parent and no grandparents.
- Grandparent-headed households (coded 2): Participants living with at least one grandparent and no parents present.
- Multi-generational households (coded 3): Participants living with at least one parent and at least one grandparent.

## 3. Results

The analyses yielded several key findings regarding the influence of caregiver type and baseline nutrition on adolescent health behaviors over time. Notably, caregiver type was significantly associated with differing trajectories for both individual exercise (*F*(6, 10,395.601) = 2.795, *p* = 0.010) and sedentary behavior (*F*(6, 18,951.310) = 23.026, *p* < 0.001). Specifically, adolescents in grandparent-only households reported higher individual exercise at baseline compared to other groups. Furthermore, baseline nutritional quality significantly predicted trajectories of individual exercise into young adulthood (Time × Nutrition interaction: *F*(2.961, 13,096.103) = 3.974, *p* = 0.012). While overall baseline dietary patterns did not significantly differ by caregiver type (Wilks’ Λ = 0.998, *p* = 0.389), an exploratory analysis revealed lower dairy consumption in homes with a grandparent present (*t*(6258) = 1.995, *p* = 0.046). The detailed results for each hypothesis are presented below.

Hypothesis 1: Caregiver Type and Baseline Nutrition: A multivariate analysis of variance (MANOVA) was conducted using pooled results from 20 imputed datasets to determine the relation between caregiver type and childhood nutrition levels at Time 1. The independent variable, caregiver type, included three levels: parents-only, grandparents-only, and multi-generational caregivers. The dependent variables consisted of five nutrition-related items: (1) consumption of dairy products; (2) consumption of fruits/fruit juice; (3) consumption of vegetables; (4) consumption of breads/pastas/rices; and (5) consumption of pastry products. Each nutrition-related item was measured on a scale from 1 (“Did not eat yesterday”) to 3 (“Ate 3 or more times yesterday”).

The pooled MANOVA results indicated no statistically significant overall association between caregiver type and the combined nutritional intake variables, Wilks’ Λ = 0.998, *F*(10, 12,978) = 1.060, *p* = 0.389, multivariate partial η^2^ = 0.001. The very small effect size (partial η^2^ = 0.001) suggests that caregiver type accounted for less than 1% of the variance in overall baseline nutritional intake. Due to the non-significant multivariate finding, follow-up univariate ANOVAs were not pursued for this primary hypothesis.

**Figure 1:**
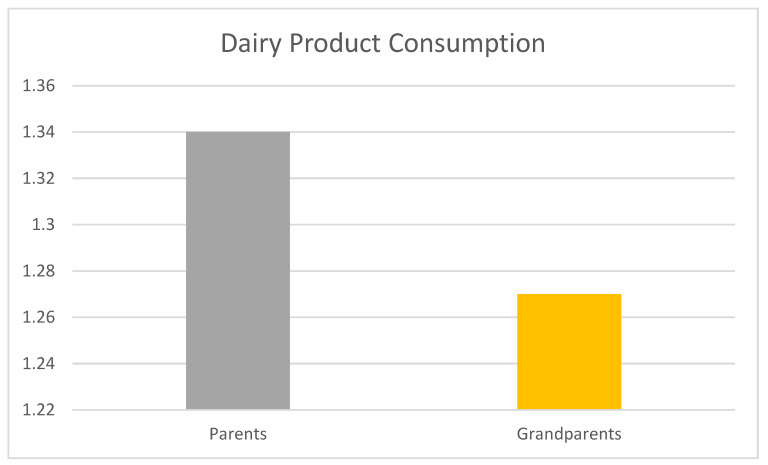
Mean adolescent dairy product consumption in parents-only vs. grandparent-present homes. Note: Mean dairy product consumption scores at Wave 1 by caregiver configuration. Scores range from 1 (“Did not eat yesterday”) to 3 (“Ate three or more times yesterday”). “Parents-Only” indicates adolescents raised by only biological or adoptive parents. “Grandparent-Present” includes both grandparent-only and multi-generational households. Values reflect pooled means from a multiply imputed dataset (N = 6260). A *t*-test revealed a statistically significant but small difference between groups; *t*(6258) = 1.995, *p* = 0.046, Cohen’s d = 0.097.

Given the non-significant main finding, an exploratory analysis focused on dairy consumption, an item often highlighted in caregiving literature [37,38]. The grandparents-only (pooled *M* = 1.20 for dairy) and multi-generational (pooled *M* = 1.30 for dairy) caregiver groups were collapsed into a single “grandparent-present” category to enhance statistical power for this exploration. An independent-samples *t*-test (using pooled results) compared dairy consumption between adolescents in “parents-only” homes and those in “grandparent-present” homes. This exploratory test revealed a statistically significant, albeit small, difference: adolescents in parents-only homes reported slightly more frequent dairy consumption (*M* = 1.34, *SD* = 0.749) than those in homes with a grandparent present (*M* = 1.27, *SD* = 0.785); *t*(6258) = 1.995, *p* = 0.046, 95% CI for mean difference [0.001, 0.144], Cohen’s d = 0.097. This comparison is illustrated in Figure 1.

Hypothesis 2A: Caregiver Type and Sedentary Activity Trajectories: To test whether adolescents’ trajectories of sedentary activity varied by caregiver type (parents-only, grandparents-only, or multi-generational households), we conducted a linear mixed-effects model using pooled results from M = 20 multiply imputed datasets. The model predicted the sedentary score across the four waves. Fixed effects included Wave (Time: Waves 1–4, with Wave 4 as the reference), Caregiver Group (1 = parents-only; 2 = grandparents-only; 3 = multi-generational, with multi-generational as the reference category), and the Wave × Caregiver Group interaction. Covariates included baseline age (H1GI1Y), sex (BIO_SEX), and race/ethnicity (H1GI8). Participant ID (AID) was included as a random intercept to account for the non-independence of repeated observations; the variance for this random intercept was statistically significant (Estimate = 2.663; SE = 0.707, *p* < 0.001), indicating significant between-participant variability in sedentary activity intercepts.

The analysis of fixed effects (Type III tests) revealed a significant main effect of Wave, *F*(3, 18,951.310) = 2799.100, *p* < 0.001. Parameter estimates indicated that on average, sedentary activity levels were significantly higher at earlier waves compared to Wave 4. Specifically, scores were higher at Wave 1 (B = 14.148; SE = 0.269, *t*(17,103.848) = 52.525, *p* < 0.001), Wave 2 (B = 12.500; SE = 0.265, *t*(16,080.304) = 47.144, *p* < 0.001), and Wave 3 (B = 11.000; SE = 0.267, *t*(16,772.097) = 41.199, *p* < 0.001) when contrasted with Wave 4, suggesting a general decrease in sedentary behavior from adolescence into young adulthood.

There was also a significant main effect of Caregiver Group, *F*(2, 6463.554) = 42.026, *p* < 0.001. Compared to adolescents in multi-generational households (reference group), those in parents-only households reported significantly lower average sedentary activity scores (B = −4.812; SE = 0.718, *t*(6497.105) = −6.704, *p* < 0.001). Similarly, adolescents in grandparents-only households also reported significantly lower average sedentary activity scores compared to the multi-generational group (B = −6.780; SE = 0.839, *t*(5590.306) = −8.079, *p* < 0.001).

**Figure 2:**
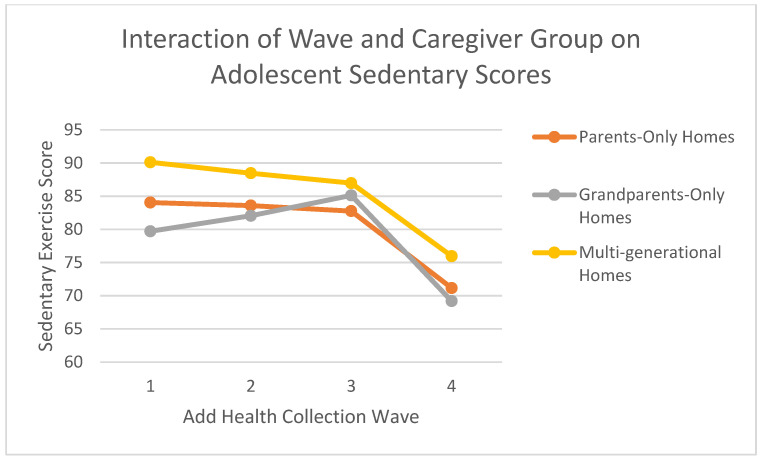
Sedentary scores across add health waves by caregiver household type. Note: Estimated marginal means of sedentary activity across Waves 1–4 by caregiver type (parents-only, grandparents-only, multi-generational). Scores reflect average hours of TV/video watching reported on weekdays and weekends, using a 6-point scale (0 = None to 5 = 5+ hours/day). Values are predicted from a linear mixed-effects model including time (Wave), caregiver type, and their interaction, with age, sex, and race as covariates. Estimates are based on pooled results from 20 multiply imputed datasets using Add Health data.

Most importantly for this hypothesis, the Wave × Caregiver Group interaction was statistically significant, *F*(6, 18,951.310) = 23.026, *p* < 0.001 (see Figure 2). This indicates that the trajectories of sedentary activity over time differed significantly by caregiver type. To explore this interaction, we examined the parameter estimates. For example, the specific interaction term for being in Wave 3 (compared to Wave 4) and in a grandparent-only household (coded 2, compared to multi-generational households) was statistically significant (B = 4.940; SE = 1.111, *t*(18,405.454) = 4.445, *p* < 0.001). This positive coefficient suggests that the sedentary activity score for the grandparent-only group at Wave 3 was 4.94 units higher than what would be expected if only the main effects of Wave 3 and the grandparent-only group were considered (relative to the reference groups Wave 4 and multi-generational).

Hypothesis 2B: Caregiver Type and Individual Exercise Trajectories: A linear mixed model (LMM) assessed the influence of caregiver type on adolescents’ time spent in individual exercise activities across four waves of data collection. Caregiver type (parents-only, grandparents-only, or multi-generational homes) was the between-subjects factor, and individual exercise activity rates (Wave) served as the within-subjects factor. The analysis included age (H1GI1Y), biological sex (BIO_SEX), and race (H1GI8) as covariates; all covariates were significantly associated with individual exercise (all *p*s < 0.001). The Satterthwaite approximation was used to estimate degrees of freedom for the fixed effects.

The LMM revealed significant within-subjects effects. Specifically, a significant main effect of time indicated that overall individual exercise levels changed across the four waves (*F*(3, 10,395.601) = 6.484, *p* < 0.001, partial η^2^ ≈ 0.002).

Further, a significant Time × Caregiver Type interaction effect emerged (*F*(6, 10,395.601) = 2.795, *p* = 0.010, partial η^2^ ≈ 0.002). This interaction signifies that the trajectories of individual exercise over time differed by caregiver type.

Regarding between-subjects effects, the main effect of caregiver type on individual exercise, when averaged across time points, was not statistically significant (*F*(2, 4470.028) = 0.719, *p* = 0.487, partial η^2^ ≈ 0.0003). Given the non-significant main effect, follow-up pairwise comparisons for overall differences between caregiver groups were not further examined.

**Figure 3:**
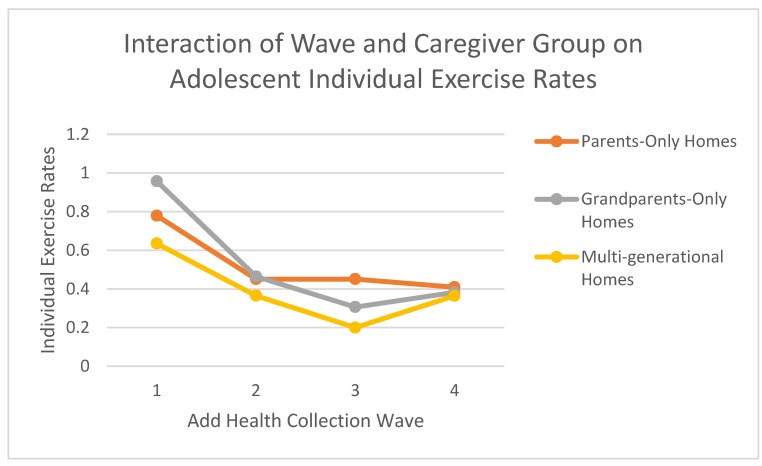
Individual exercise rates across add health waves by caregiver household type. Note: Estimated marginal means of individual exercise across Waves 1–4 by caregiver type (parents-only, grandparents-only, multi-generational). Individual exercise was assessed via reported hours of solo physical activity (e.g., jogging, yoga) on weekdays and weekends, using a 6-point scale (0 = None to 5 = 5+ hours/day). Estimates were generated from a linear mixed-effects model that included fixed effects for wave, caregiver group, and their interaction, adjusting for age, sex, and race. Results reflect pooled data from 20 multiply imputed datasets (Add Health study).

To explore the significant Time × Caregiver type interaction, we conducted follow-up pairwise comparisons using an LSD (least significant difference) adjustment, examining differences in estimated marginal means for Individual Exercise:

- At Time 1 (Wave 1) (see Figure 2):
  ○ Adolescents in parent-only households had an estimated mean of 0.779 (SE = 0.020).
  ○ Adolescents in grandparent-only households had an estimated mean of 0.957 (SE = 0.086).
  ○ Adolescents in multi-generational households had an estimated mean of 0.635 (SE = 0.057).
  ○ Pairwise comparisons revealed that adolescents in grandparent-only households reported significantly higher individual exercise than those in parent-only households (mean difference = 0.178; SE of difference = 0.088, *p* = 0.043) and significantly higher than those in multi-generational households (mean difference = 0.322; SE of difference = 0.103, *p* = 0.002). Additionally, adolescents in parent-only households reported significantly higher individual exercise than those in multi-generational households (mean difference = 0.144; SE of difference = 0.060, *p* = 0.017).

- At Time 3 (Wave 3) (see Figure 3):
  ○ Adolescents in parent-only households had an estimated mean of 0.451 (SE = 0.020).
  ○ Adolescents in grandparent-only households had an estimated mean of 0.306 (SE = 0.086).
  ○ Adolescents in multi-generational households had an estimated mean of 0.200 (SE = 0.058).
  ○ Pairwise comparisons revealed that adolescents in parent-only households reported significantly higher individual exercise than those in multi-generational households (mean difference = 0.251; SE of difference = 0.061, *p* < 0.001). The difference between parent-only and grandparent-only households was not statistically significant at this wave (mean difference = 0.145; SE of difference = 0.089, *p* = 0.103), nor was the difference between grandparent-only and multi-generational households (mean difference = 0.106; SE of difference = 0.104, *p* = 0.306).

Hypothesis 2C: Caregiver Type and Team Sport Participation Trajectories: To examine the influence of caregiver type on adolescents’ time spent in team sports participation over time, a linear mixed model (LMM) was conducted. Caregiver type (parents-only, grandparents-only, or multi-generational homes) served as the between-subjects factor, and team sports participation rates (Wave) served as the within-subjects factor. Covariates included adolescent age, biological sex, and race. The Satterthwaite approximation was used for degrees of freedom.

The LMM revealed a significant main effect of time, indicating that team sports participation changed significantly across the four waves overall (*F*(3, 10,636.671) = 12.512, *p* < 0.001, partial η^2^ ≈ 0.004). However, the Time × Caregiver Type interaction effect was not statistically significant (*F*(6, 10,636.671) = 0.723, *p* = 0.631, partial η^2^ ≈ 0.0004). This indicates that the trajectories of team sports participation over time did not significantly differ by caregiver type.

The between-subjects main effect of caregiver type was also not statistically significant (*F*(2, 4607.494) = 0.816, *p* = 0.442, partial η^2^ ≈ 0.0004), suggesting no significant difference in average team sports participation across the three caregiver groups when collapsed across time. Covariates of age (*F*(1, 4521.904) = 816.164, *p* < 0.001), biological sex (*F*(1, 4529.130) = 289.184, *p* < 0.001), and race (*F*(4, 5526.628) = 4.717, *p* = 0.001) were all significantly associated with team sports participation.

Hypothesis 3A: Influence of Baseline Nutrition on Sedentary Activities Over Time: To examine the influence of baseline nutrition level on adolescents’ sedentary activity rates over time, a general linear model (GLM) repeated-measures analysis was conducted. Nutrition level (dichotomized as high versus low based on Wave 1 dietary intake) served as the between-subjects factor, and sedentary activity rates across the four waves of data collection served as the within-subjects factor. Covariates included age, biological sex, and race.

The assumption of sphericity was tested using Mauchly’s test of sphericity, which indicated that the assumption was violated (χ^2^(5) = 747.199, *p* < 0.001). Therefore, the Greenhouse–Geisser correction was used to adjust the degrees of freedom for within-subjects effects.

The GLM revealed a significant main effect of time, indicating that sedentary activity levels changed significantly across the four waves overall (*F*(2.622, 11,597.106) = 1288.299, *p* < 0.001, partial η^2^ = 0.226, using Greenhouse–Geisser correction). However, the Time × Nutrition Level interaction effect was not statistically significant (*F*(2.622, 11,597.106) = 1.529, *p* = 0.214, partial η^2^ = 0.000, using Greenhouse–Geisser correction). This indicates that the change in sedentary behavior over the four waves did not significantly differ between adolescents classified with low versus high nutritional intake at Wave 1.

Examining the between-subjects effects, the main effect of nutrition level was not statistically significant (*F*(1, 4422) = 0.037, *p* = 0.847, partial η^2^ = 0.000), suggesting no significant difference in average sedentary activity levels between the low and high nutrition groups when collapsed across time. The covariates of age (*F*(1, 4422) = 10.370, *p* = 0.001, partial η^2^ = 0.002), biological sex (*F*(1, 4422) = 6.761, *p* = 0.009, partial η^2^ = 0.002), and race (*F*(4, 4422) = 7.146, *p* < 0.001, partial η^2^ = 0.006) were all significantly associated with sedentary activities.

Hypothesis 3B: Influence of Baseline Nutrition on Individual Exercise Activities Over Time: To examine the influence of baseline nutrition level on adolescents’ time spent in individual exercise activities across waves, a general linear model (GLM) repeated-measures analysis was conducted. Nutrition level (dichotomized as high versus low based on Wave 1 dietary intake) served as the between-subjects factor, and individual exercise rates across the four waves of data collection served as the within-subjects factor. Covariates included age, biological sex, and race. The assumption of sphericity was tested using Mauchly’s test of sphericity, which indicated a violation (χ^2^(5) = 56.423, *p* < 0.001). Therefore, the Greenhouse–Geisser correction was applied to the degrees of freedom for within-subjects effects (ε = 0.987).

The GLM revealed significant within-subjects effects. There was a significant main effect of time, indicating that individual exercise levels changed significantly across the four waves overall (*F*(2.961, 13,096.103) = 1248.950, *p* < 0.001, partial η^2^ = 0.221, using Greenhouse–Geisser correction). More central to Hypothesis 3B, there was a significant Time × Nutrition level interaction effect (*F*(2.961, 13,096.103) = 3.974, *p* = 0.012, partial η^2^ = 0.001, using Greenhouse–Geisser correction). This significant interaction indicates that the trajectories of individual exercise participation over time differed significantly based on baseline nutrition level.

Examining the between-subjects effects, there was also a significant main effect of nutrition level (*F*(1, 4423) = 10.550, *p* = 0.001, partial η^2^ = 0.002). Averaged across the four waves, adolescents classified in the high nutrition group at baseline reported significantly higher individual exercise (estimated marginal mean = 0.686; SE = 0.025) compared to those in the low nutrition group (estimated marginal mean = 0.581; SE = 0.027). The covariates of age (*F*(1, 4423) = 11.714, *p* = 0.001, partial η^2^ = 0.003), biological sex (*F*(1, 4423) = 159.612, *p* < 0.001, partial η^2^ = 0.035), and race (*F*(4, 4423) = 7.575, *p* < 0.001, partial η^2^ = 0.007) were also significantly associated with individual exercise.

**Figure 4:**
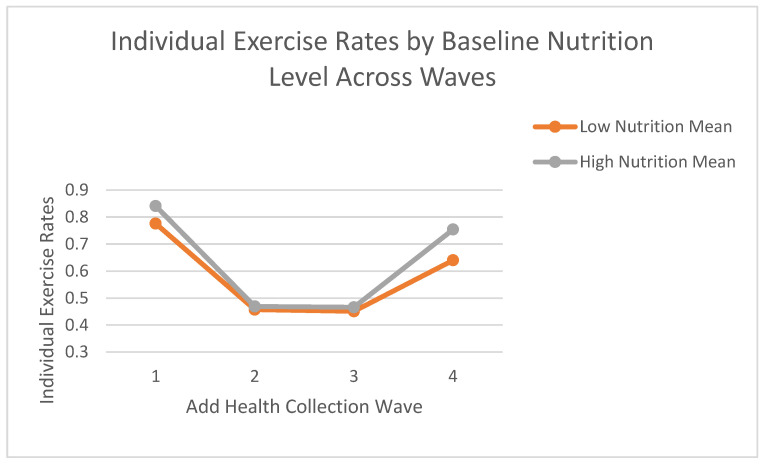
Mean individual exercise rates by baseline nutrition level across waves. Note: Estimated marginal means of individual exercise rates across Waves 1–4 by baseline nutrition group. Baseline nutrition was categorized as “low” (≤3 healthy food categories consumed on the previous day) or “high” (>3). Individual exercise was self-reported on a 6-point scale (0 = None to 5 = 5 or more hours/day), averaged across weekdays and weekends. Means are derived from a general linear model with nutrition group as the between-subjects factor and wave as the within-subjects factor, adjusting for age, sex, and race. Values reflect pooled estimates from 20 multiply imputed datasets using Add Health data.

Given the significant Time × Nutrition level interaction, follow-up Bonferroni-adjusted pairwise comparisons were conducted to examine differences between nutrition levels at each time wave. These comparisons revealed that at Time 4 (Wave 4), adolescents in the high nutrition group (estimated marginal mean = 0.754; SE = 0.025) reported significantly higher individual exercise compared to those in the low nutrition group (estimated marginal mean = 0.640; SE = 0.027; mean difference = 0.114; SE of difference = 0.034, Bonferroni-adjusted *p* = 0.005; see Figure 4). No other pairwise comparisons between nutrition groups at Waves 1, 2, or 3 were statistically significant (all Bonferroni-adjusted *p*s > 0.05). These results suggest that baseline nutritional intake has a lasting association with individual exercise patterns, with differences between groups emerging in young adulthood (Wave 4).

Hypothesis 3C: Influence of Baseline Nutrition on Team Sports Participation Over Time: To examine the influence of baseline nutrition level on adolescents’ time spent in team sports participation over time, a general linear model (GLM) repeated-measures analysis was conducted. Nutrition level (dichotomized as high versus low based on Wave 1 dietary intake) served as the between-subjects factor, and team sports participation rates across the four waves of data collection served as the within-subjects factor. Covariates included age, biological sex, and race.

Mauchly’s test of sphericity indicated that the assumption of sphericity was violated (χ^2^(5) = 28.717, *p* < 0.001). Therefore, the Greenhouse–Geisser correction was used to adjust the degrees of freedom for within-subjects effects (ε = 0.991).

The GLM revealed a significant main effect of time, indicating that team sports participation levels changed significantly across the four waves overall (*F*(2.972, 13,144.600) = 1125.868, *p* < 0.001, partial η^2^ = 0.203, using Greenhouse–Geisser correction). However, the Time × Nutrition level interaction effect was not statistically significant (*F*(2.972, 13,144.600) = 1.074, *p* = 0.359, partial η^2^ = 0.000, using Greenhouse–Geisser correction). This indicates that the change in team sports participation over the four waves did not significantly differ between adolescents classified with low versus high nutritional intake at Wave 1.

Examining the between-subjects effects, the main effect of nutrition level was not statistically significant (*F*(1, 4422) = 0.050, *p* = 0.824, partial η^2^ = 0.000), suggesting no significant difference in average team sports participation levels between the low and high nutrition groups when collapsed across time. The covariates of age (*F*(1, 4422) = 12.922, *p* < 0.001, partial η^2^ = 0.003), biological sex (*F*(1, 4422) = 195.202, *p* < 0.001, partial η^2^ = 0.042), and race (*F*(4, 4422) = 2.951, *p* = 0.019, partial η^2^ = 0.003) were all significantly associated with team sports participation.

## 4. Discussion

### 4.1. Summary of Key Findings in a Socioecological Context

This longitudinal analysis of Add Health data revealed modest yet noteworthy associations between family caregiver structure during adolescence, and both nutritional and physical activity patterns that extend into young adulthood. Specifically, grandparent involvement in caregiving was linked to lower adolescent dairy intake compared to parent-only homes, although overall dietary patterns did not significantly differ by caregiver type. In contrast, youth raised exclusively by grandparents demonstrated consistently higher engagement in individual physical activity over time compared to their peers in parent-only or multi-generational households. No significant differences by caregiver group emerged for sedentary behaviors or team sports participation. It is important to note, however, that the observed effect sizes for some of these findings were small (e.g., partial η^2^ ≈ 0.002 for the time x caregiver type interaction on individual exercise; partial η^2^ = 0.001 for the overall association between caregiver type and combined nutritional intake), suggesting a need for interpretative caution. A key finding was that adolescents’ baseline nutritional quality significantly predicted their individual exercise levels in young adulthood, regardless of caregiver household type, highlighting the lasting influence of early diet on later activity.

Framed within Bronfenbrenner’s socioecological model [8,9], these results underscore the nuanced role of the family microsystem (the immediate caregiving environment) and the chronosystem (the influence of time and life transitions) in shaping health behaviors. Variations in the microsystem, such as being raised by parents alone versus with grandparents, showed a limited impact on broad dietary intake. However, these variations did influence specific dietary components (e.g., dairy) and long-term individual exercise trajectories. The connection between early nutrition and subsequent exercise patterns exemplifies chronosystem effects, wherein early-life exposures within the home environment exert lasting effects as adolescents mature. Taken together, these results suggest that while many factors influence core diet and activity habits, the composition of the home caregiving environment can leave a measurable, albeit sometimes modest, imprint on health behavior well into adulthood.

### 4.2. Nutrition-Related Findings

The finding that adolescents in homes with grandparent caregivers showed lower dairy intake both aligns with and diverges from previous research, which has presented mixed results on the impact of grandparent caregiving on child health behaviors [39]. The observed lower dairy consumption resonates with some studies suggesting that grandparent-provided diets may not always align with optimal nutritional balance. For instance, research indicates that grandparents might sometimes indulge grandchildren with energy-dense “treat” foods, such as sweetened drinks, potentially displacing more nutritious options like dairy products [16,17,40]. Such indulgent feeding, aimed at expressing affection, has been linked to higher obesity risk in children raised by grandparents [16]. This could explain the modest but significant difference in dairy consumption found in our study (comparing “parents-only” to “grandparent-present” homes, which combined grandparents-only and multi-generational households for this specific exploratory analysis). This could be due to generational dietary habits, economic limitations, or outdated nutritional knowledge. Indeed, a prior study noted more frequent unhealthy snacking and even hunger in skipped-generation families compared to multi-generational homes, emphasizing potential inconsistencies in food availability and quality in some grandparent-led households [17]. It should also be noted that children, especially adolescents, are less likely to consume milk and dairy though they consume cheese from other products in general [41]. Our findings on dairy might be a subtle indicator of such dietary quality variations.

However, the overall dietary patterns showed an absence of broader dietary differences across caregiver types in our main analysis. Adolescents report consuming vegetables, fruits, grains and pastries at the same rate in parent-only and grandparent-present households. Grandparent caregiving is not uniformly detrimental to children’s overall nutrition. This aligns with other research highlighting positive nutritional influences from grandparents, such as providing home-cooked meals or encouraging fruit and vegetable intake [16]. Grandparents in caregiving roles have reported many similar feeding practices to parents, including regular vegetable provision [40], although these may coexist with more permissive approaches like offering food-based rewards [16,40]. Overall, these nutrition-related results contribute to the complex picture in existing literature; grandparent caregiving can sometimes be associated with less healthy eating habits, but its impact is not universally negative and depends heavily on individual and contextual factors.

### 4.3. Physical Activity Findings

Our most striking physical activity result was that adolescents raised in homes headed by grandparents without parents present exhibited higher rates of individual exercise participation over time. This finding was contrary to our Hypothesis 2B, which predicted greater individual exercise in parent-headed homes without grandparents present, and adds a novel dimension to the literature that has often reported mixed or negative outcomes for physical activity under grandparent care [18,24]. Some prior studies have raised concerns that children raised by grandparents may lead more sedentary lifestyles or exceed screen-time limits [42], often attributed to grandparents’ permissive styles or physical limitations [22,43].

However, other research aligns more closely with our findings, suggesting grandparent involvement can sometimes increase children’s activity levels. For instance, a study of Latino families found co-residence with grandparents associated with a greater likelihood of children achieving daily physical activity recommendations, despite also noting higher screen time [18]. Qualitative studies also describe both barriers and contributors (e.g., more time for supervision) to supporting grandchildren’s active play in grandparent-led homes [24]. The significant gap in individual exercise we observed (evident from Wave 1 means for grandparent-only households (e.g., M = 0.957 at Wave 1) compared to parent-only (M = 0.779 at Wave 1) or multi-generational homes (M = 0.635 at Wave 1), with trajectories diverging over time) warrants careful consideration of potential mechanisms. Adolescents in grandparent-only homes might develop greater self-reliance for physical activity, engaging in independent outdoor play or physical chores, especially if older caregivers are less able to participate in or transport them to structured activities. The absence of a similar advantage in multi-generational homes suggests parental presence might moderate grandparental influence, perhaps through differing activity priorities or intergenerational disagreements on child-rearing [39]. The consistently higher individual exercise in grandparent-only homes implies these households may foster environments supportive of solitary activities like jogging or at-home exercises, which require less direct parental involvement or financial investment in equipment and fees. This socioecological microsystem perspective suggests that specific family structures (grandparent-only) may cultivate certain positive behaviors (individual exercise) even if sometimes viewed as risk environments for others. Our data do not suggest grandparent care is universally “better” for physical activity but highlight how different contexts may facilitate different activity types.

### 4.4. Potential Explanations for Null Findings for Sedentary Activities and Team Sports Participation

Caregiver type did not significantly impact adolescents’ trajectories of sedentary behavior or team sports involvement. Several factors could explain these null findings. First, broader social and environmental factors, such as peer influence, school environments, and community opportunities, may more strongly govern these specific activity domains than the immediate family caregiving structure. By adolescence, sedentary screen use is often ubiquitous, driven by technology access and youth culture, potentially diminishing detectable differences across household types over time [42].

Second, participation in team sports heavily depends on access to school programs, community leagues, and socioeconomic resources, which might overshadow caregiver-type effects. While we hypothesized grandparents might face more barriers to supporting organized sports [22], unmeasured socioeconomic factors could have masked true differences. Grandparent-headed households often report greater socioeconomic disadvantages [44,45], potentially reducing youth sports participation. However, if some parent-only families faced similar barriers, this could result in no net difference between groups. Our lack of detailed socioeconomic controls limits definitive conclusions here.

Third, measurement limitations are a consideration. Self-reported sedentary behavior and physical activity are prone to recall bias. Additionally, the broad nature of the “team sports” measure might not capture qualitative differences in engagement. Such methodological factors could obscure subtle effects. Finally, it is plausible that caregiver structure exerts a more pronounced impact on specific health behaviors (like diet and individual exercise) than on others like team sports or ubiquitous sedentary pastimes, which may be more influenced by factors beyond direct caregiver control or where different types of caregivers exhibit similar encouragement or permission behaviors.

### 4.5. Public Health and Clinical Implications

These findings carry important public health implications, especially for addressing adolescent obesity and promoting healthy lifestyles across diverse family settings. The observation that caregiver type can shape long-term exercise trajectories and specific dietary habits, even if some effects are modest, highlights the need to broaden nutrition and physical activity interventions beyond the traditional parent-child dyad. Recognizing that adolescents raised by grandparents may have lower dairy intake but higher individual exercise suggests a need for tailored support. For instance, the statistically significant, albeit small, difference in dairy consumption (Cohen’s d = 0.097) between parent-only and grandparent-present homes, if indicative of lower calcium intake, could have clinical significance for bone health development during adolescence, a critical period for bone mass accrual. Similarly, the higher individual exercise rates in grandparent-only households, if sustained, offer a potential protective factor.

Public health interventions should ensure grandparent caregivers access up-to-date nutrition education (e.g., on current dairy recommendations) and resources. Involving grandparents in health promotion can be beneficial [16,18,45]. Community nutrition workshops and pediatric health counseling should explicitly include grandparent guardians, equipping them with strategies for current dietary guidelines and encouraging active play. Family-centered interventions involving multiple generations should leverage each caregiver’s strengths and promote consistent health messages to avoid intergenerational conflict regarding food or activity, which can negatively impact child outcomes [15,39]. Policies improving grandparent-led families’ access to food assistance or affordable youth sports programs could also mitigate health disparities linked to economic strain often experienced by these households [22,23].

### 4.6. Strengths and Limitations

Our focus on grandparent-only and multi-generational homes addresses an understudied and emerging dimension for adolescent and family health. This study’s strengths include its use of a longitudinal, nationally representative dataset (Add Health), allowing us to track behavior patterns from adolescence into young adulthood across a diverse sample, enhancing generalizability. Controlling covariates and examining trajectories over four waves strengthens potential causal inferences compared to cross-sectional designs. However, several limitations warrant a critical discussion, urging caution in interpreting the findings. Self-report bias is a significant concern for both dietary intake (relying on “yesterday’s” recall of broad food categories) and physical activity measures, potentially underestimating or overestimating true behaviors. The dietary measures were limited, lacking detailed nutrient intake, which may obscure finer diet quality differences. It is unclear, for example, if cheese on pizza was considered as a measurement of dairy intake. While the study included longitudinal outcomes, caregiver type and baseline diet were assessed only at Wave 1, rendering those aspects of the analysis effectively cross-sectional for predicting trajectories. Changes in living situations over the 14-year span were not accounted for, potentially misclassifying caregiver type in later waves and biasing results toward the null.

Another limitation is the lack of detailed socioeconomic data (e.g., income, education) and other potential confounders in our models. Grandparent-led households often differ systematically from parent-led homes in these aspects [20,22], and these unmeasured factors may additionally explain observed differences attributed caregiver type. The relatively small number of adolescents in the grandparents-only group also limits statistical power and the precision of estimates for this specific group, potentially leading to undetected effects or unstable findings. The modest effect sizes for some significant findings (e.g., partial η^2^ values often ≤ 0.002) indicate that while statistically significant, the clinical or practical significance of these specific differences might be small for some outcomes.

Finally, this study focused on behaviors, not direct health endpoints like BMI or disease incidence. Wearable devices could objectively monitor physical activity and sedentary time. Comprehensive dietary assessments (e.g., 24-h recalls, food frequency questionnaires) would provide more detailed intake data, using methods that are less reliant on long recall. Expanding outcomes to include health indices (BMI, blood pressure) would help determine if behavioral differences translate into meaningful health differences. Therefore, while we infer potential health implications, we cannot confirm whether observed behavioral differences translated into differential health outcomes. Further research in this area should consider including these methods and outcomes to provide this conclusion.

### 4.7. Future Directions

Our findings open several avenues for future research. Qualitative studies are needed to explore the mechanisms underlying the observed associations to understand food purchasing, meal routines, and physical activity encouragement in grandparent-led households. This could clarify overall attitudes and practices towards overall dietary practices and what grandparents want to know and how they want to learn it. Research in varied cultural and geographic contexts is also needed, as grandparenting roles and health behaviors can differ. Given that the Add Health Wave 1 data are from the mid-1990s, contemporary studies are essential to assess if these trends persist in today’s digital and evolving family landscape.

Future studies should examine the role of specific grandparenting styles (e.g., authoritarian vs. indulgent), health status, and parenting philosophies, as these likely mediate child outcomes [46]. More detailed comparisons between multi-generational and skipped-generation households are warranted, as well as research into the interplay between parents and grandparents in multi-generational homes. Longitudinal research tracking changes in family composition would highlight chronosystem effects more dynamically.

Finally, nutrition education and intervention trials tailored for grandparent caregivers are needed. Building an evidence base for family-centric health solutions will require intentional understanding of the complexity of these types of households and effective research design.

## 5. Conclusions

This study suggests that the composition of the adolescent caregiving environment is associated with certain long-term health-related behaviors, although some of these associations are modest. For the growing segment of youth raised by grandparents, our findings indicate potential links to subtle dietary differences, such as lower dairy intake, and notably, to higher rates of individual physical activity extending from adolescence into young adulthood. Furthermore, early nutritional habits appear to be an important predictor of later individual exercise, underscoring how behaviors established within the family microsystem may resonate across the chronosystem of development [8]. The lack of significant variation in sedentary time and team sports participation by caregiver type suggests these aspects of lifestyle may be more strongly influenced by factors outside the immediate family context, or that different caregiver types may offer comparable support or limitations in these specific domains.

The findings, while requiring cautious interpretation due to study limitations and the magnitude of some effects, have practical implications. They underscore the importance of recognizing and supporting diverse family structures within public health initiatives focused on nutrition and physical activity. Family-based interventions aimed at mitigating adolescent obesity and promoting active lifestyles should consider grandparents as potentially vital influencers. Engaging entire family units, including various caregiving configurations, may be crucial for effectively nurturing healthy behaviors in younger generations. By acknowledging the complexities of family dynamics and tailoring strategies to meet the needs of non-traditional caregivers, public health efforts can better support all children—regardless of whether they are raised by parents, grandparents, or in multi-generational settings—in building a foundation for active and well-nourished lives that extend into adulthood. This research contributes to the understanding that the broader caregiving “village” surrounding a child can indeed shape their health journey, warranting its consideration in the design of future research and interventions.

## Data Availability

Publicly available datasets were analyzed in this study. These data can be found here: https://addhealth.cpc.unc.edu/.
